# Aqueous root extract of *Asparagus cochinchinensis* (Lour.) Merr. Has antioxidant activity in D-galactose-induced aging mice

**DOI:** 10.1186/s12906-017-1975-x

**Published:** 2017-09-25

**Authors:** Linghua Lei, Yanhua Chen, Lijun Ou, Yinglong Xu, Xiaoying Yu

**Affiliations:** 1 College of Ecology, Lishui University, Lishui, Zhejiang, 323000 China; 2 College of Horticulture and Landscape, Hunan Agriculture University, Changsha, Hunan 410128 China; 3 Vegetable Research lnsitute, Agricultural Sciences Academy of Hunan Provincial, Changsha, Hunan 410125 China; 4 Hunan Institute of Science and Technology, Yueyang, Hunan 414006 China

**Keywords:** *Asparagus cochinchinensis* (Lour.) Merr., Antioxidant, Aging, Enzyme activity, Root

## Abstract

**Background:**

Extracts of plants have been considered as sources of natural antioxidant agents. In this study, we aimed to explore the antioxidant capacity of the aqueous root extract of *Asparagus cochinchinensis* (Lour.) Merr.

**Methods:**

Using vitamin C (Vc) as a positive control, we analyzed the aqueous root extract of *A. cochinchinensis* free radical scavenging ability *in vitro*. We also established a mouse aging model using D-galactose and then treated it with aqueous root extract or Vc. The blood cell count and superoxide dismutase (SOD), catalase (CAT), and nitric oxide synthase (NOS) activities as well as malondialdehyde (MDA) and nitric oxide (NO) contents were measured; pathological examination of tissues was performed; and SOD, glutathione peroxidase (GPX), and NOS expression levels in the serum, liver, and brain tissues were investigated.

**Results:**

*In vitro*, compared with the antioxidant Vc, the aqueous root extract showed similar 1,1-Diphenyl-2-picrylhydrazyl radical and 3-ethylbenzothiazoline-6-sulfonic·scavenging activities and even significantly increased superoxide anion (*p* < 0.05) and hydroxyl radical (OH) (*p* < 0.01) scavenging activities. The aqueous extract significantly increased the white blood cell count as well as enhanced SOD, CAT, and NOS activities (*p* < 0.01) in aging mice. In addition, the aqueous extract increased the NO content (*p* < 0.05) and reduced the MDA content (*p* < 0.05).

**Conclusions:**

The aqueous root extract of *A. cochinchinensis* showed as strong antioxidant ability as Vc and might prevent aging by reducing radicals.

## Background

Aging, which refers to a multidimensional process of physical and psychological changes, is closely related to most human diseases [1]. Aging is associated with reduced antioxidant enzyme, such as superoxide dismutase (SOD), activities, thereby attenuating the removal of oxygen radicals [2]. Currently, exogenous radical scavengers, such as butyl hydroxy toluene, butylated hydroxylanisole, and tert-butylhydroquinone, have been successfully used for resisting disease development [3]. However, the toxicity of artificial antioxidants can lead to risks of DNA damage and malignancy [4]. Thus, the safety of artificial antioxidants remains a subject of debate [5].

Natural antioxidants have attracted attention as replacements for artificial antioxidants, [6]. Okra leaf [7] and *Rubus alceifolius* Poir [8], which are traditional Chinese medicines, are deemed to be potential resources of natural antioxidants. *Asparagus cochinchinensis* (Lour.) Merr. *(A. cochinchinensis)*, one of the medicinally important plants, has antibacterial, anti-inflammatory, anticancer, and antioxidant effects [9–12]. A methanol extract from *A. cochinchinensis* has a neuroprotective effect in cerebral infarction model animals [13]. Our recent study also demonstrated the antioxidant ability of shoot extract of *A. cochinchinensis* in mice with D-galactose-induced aging [14]. However, antioxidant effects of the tuberous root of *A. cochinchinensis* remain unclear.

A previous study revealed that an aging model can be induced using D-galactose [15]. In this study, D-galactose-induced aging mouse model was established, and then the aging mice were treated with aqueous root extract or Vc. The blood cell count and superoxide dismutase (SOD), catalase (CAT), and nitric oxide synthase (NOS) activities as well as malondialdehyde (MDA) and nitric oxide (NO) contents were measured; pathological examination of tissues was performed; and SOD, glutathione peroxidase (GPX), and NOS expression levels in serum, liver, and brain tissues was investigated. In addition, we investigated the effect of the aqueous root extract of *A. cochinchinensis* on radical scavenging ability in vitro. We also aimed to explore the antioxidant mechanism and further application of the aqueous root extract of *A. cochinchinensis*.

## Methods

### Pharmaceutical preparation

The aqueous root extract was prepared as described previously [16]*. A. cochinchinensis* specimens were collected from Lewang Town, Wangmo County, Buyi and Miao Nationalities Autonomous Prefecture, Guizhou Province, and identified by Professor Wu Xian at Hunan Huaihua College. This material (voucher specimen number: HJH20150930013) was deposited in the Plant Herbarium, Institute of Biology, Guizhou Academy of Sciences. The roots of *A. cochinchinensis* were dried with hot air. After grinding, powdered roots (20 g) were dissolved in water (160 mL), and then boiled and extracted three times. The three extracts were combined, filtered, and concentrated using a rotating evaporator to obtain the aqueous extracts of *A. cochinchinensis*, in accordance with Chinese pharmacopoeia [17]. Theaqueous extracts were dissolved in distilled water to a stocking solution of 0.7 g/mL and frozen until use.

### *Measurement of radical scavenging* ability in vitro

1,1-Diphenyl-2-picrylhydrazyl radical (DPPH) has been widely used for antioxidant assays. DPPH·and 3-ethylbenzothiazoline-6-sulfonic (ABTS^+^) scavenging activities was measured in accordance with the procedure described in our previous report [14]. In brief, 2 mL of 0.7 g/mL root extract solution was reacted with 2 mL of 1.25 × 10^−4^ mol/L DPPH or 30 μL of 0.7 g/mL root extract solution was reacted with 3 mL of 7 mmol/L ABTS^+^ at room temperature in the dark, after which the absorbance was detected at 517 or 734 nm, respectively. Negative and positive controls were ethanol (solvent) and vitamin C (Vc), respectively. Furthermore, the superoxide anion and hydroxyl radical (OH) levels were measured using commercial kits (Jiangcheng Bioengineering Institute, Nanjing, China). The absorption value was detected using a microplate reader (Thermo).

### Animal models and drug treatment

Approval was obtained from the Animal Ethics Committee of the Animal Laboratory Center of Xiangya Medical School of Central South University prior to using the animals for the following experiments. In total, 80 healthy male KunMing (KM) mice, which weighed 20 ± 2 g and were aged 2 months old, were provided by the Xiangya Medical School of Central South University. Mice were randomly and equally assigned to four groups: negative control, aging model, Vc positive control, and extract treatment. Mice in the negative control group were subcutaneously injected with saline (100 mg/kg) daily. In the aging model group, aging was induced by D-galactose, in accordance with a previously described method [18]. Mice in the aging model, Vc positive control, and extract treatment groups received a subcutaneous injection of 500 mg/kg D-galactose daily; meanwhile, mice in Vc positive control or extract treatment groups were treated with Vc or aqueous root extract (200 mg/kg) daily by intragastric administration for 30 consecutive days.

### Preparation of blood and pathological tissue sample

After the final drug administration for 24 h, 20 μL blood samples were taken by eyeball extirpation and then centrifuged at 3000 r/min for 10 min. The supernatant was used for the following experiments.

The mice were killed and then their brains, hearts, kidneys, and livers were isolated. Tissues were pre-fixed in Bouin’s solution for 24 h, followed by gradient ethanol dehydration, paraffin embedding, slicing (5–7 μm), and regular HE staining. Finally, a microscope (Motic Group Co., Ltd., Xiamen, China) was used to observe these sections.

### Measurements of the NOS, SOD, and CAT activities and NO and MDA contents

Tissues (including livers, kidneys, hearts, and brains; 0.3–0.6 g) were ground. After centrifugation at 1000 r/min for 5 min, NOS, SOD, and CAT activities as well as NO and MDA contents in the supernatants were measured using commercial kits (Jiangcheng Bioengineering Institute).

### Semi-quantitative reverse-transcription polymerase chain reaction (RT-PCR)

Total mRNA of the serum was extracted using an RNA extraction kit (Ambiogen Life Science Technology Ltd.). Then, cDNA was synthesized using a first-strand cDNA synthesis kit (Tiangen Biotech Co., Ltd., Beijing, China). Primers used in the present study are shown in Table 1. PCR reaction was conducted in a 50 mm^3^ system containing 5.0 mm^3^ of 10× PCR buffer, 10 pmol forward and reverse primers, 0.3 mm^3^ of 10 mM dNTPs, 2 U Taq (Ferments, USA), and 60 ng of the template under the following conditions: 5 min at 94 °C, followed by 35 cycles of 60 s at 94 °C, 50 s at 56 °C or 67 °C, and 50 s at 72 °C and 10 min at 72 °C. PCR reactions were terminated before the reaction reached platform, and amplicons were examined using gel electrophoresis.

**Table 1:** Primer sequences for specific genes

Gene	Primer sequence
SOD	Forward: 5′-ACGAAGGGAGGTGGATGCTG-3′
	Reverse: 5′-ACGGTTGGAGGCGTTCTGCT-3′
NOS	Forward: 5′-TTGGAGCGAGTTGTGGATTG-3′
	Reverse: 5′-TGAGGGCTTGGCTGAGTGA-3′
GPX	Forward: 5′-GCCTGGATGGGGAGAAGATA-3′
	Reverse: 5′-GCAAGGGAAGCCGAGAACTA-3′
β-actin	Forward: 5′-GAGACCT TCAACACCCCAGC-3′
	Reverse: 5′- ATGTCACGCACGATTTCCC −3′

*SOD* superoxide dismutase, *NOS* nitric oxide synthase, *GPX* glutathione peroxidase

### Statistical analysis

Statistical evaluation was performed using SPSS 18.0 package (SPSS Inc., Chicago, IL, USA). All data are expressed as mean ± S.D. The t-test was used to compare differences between the two groups in the in vitro analysis. One-way analysis of variance was used for comparing differences among the four groups in the in vivo analysis. A value of *p* < 0.05 was considered significant for all tests.

## Results

### Radical scavenging ability in vitro

**Fig. 1:**
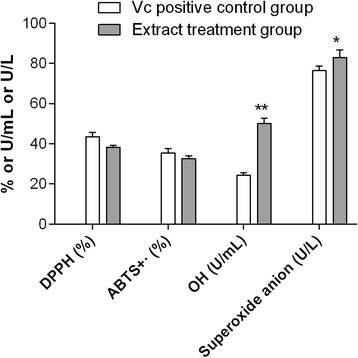
Radical scavenging ability of *Asparagus cochinchinensis* (Lour.) Merr. determined by detecting 1,1-diphenyl-2-picrylhydrazyl radical (DPPH) and 3-ethylbenzothiazoline-6-sulfonic (ABTS^+^·) scavenging activities in vitro. Experiments were repeated three times. *p < 0.05 and **p < 0.01 compared with the Vc control group

Compared with the Vc positive control group, 0.7 g/mL aqueous root extract of *A. cochinchinensis* had similar DPPH·and ABTS^+^·scavenging activities, but significantly increased superoxide anion (*p* < 0.05) and OH scavenging activities (*p* < 0.01) (Fig. 1), which suggested strong radical scavenging ability of the aqueous root extract in vitro.

### Antioxidation ability in vivo

As shown in Table 2, CAT, NOS, and SOD activities were clearly lower in the serum, kidney, heart, brain, and liver samples (*p* < 0.05) of the aging model group than in that of the negative control group. SOD, NOS, and CAT activities in the extract treatment group were elevated (*p* < 0.05) compared with those in the aging model group. No significant difference was observed in NOS, CAT, and SOD activities between the extract treatment group and Vc group. These results indicate that the aqueous root extract had similar activities for enzymes of the antioxidant system in comparison with Vc. Moreover, compared with the control group, decreased NO content and increased MDA content were observed in the aging model group (*p* < 0.05), whereas the extract of *A. cochinchinensis* significantly increased the NO content in aging mice and reduced their MDA content (Table 3). Meanwhile, NO and MDA contents were similar between extract treatment and Vc groups (Table 3).

**Table 2:** Effects of *Asparagus cochinchinensis* (Lour.) Merr on the activities of NOS, CAT and SOD

	Treatment	SOD/U mg^−1^ prot	NOS/U mg^−1^ prot	CAT/U mg^−1^ prot
Brain	Negative control group	98.31 ± 4.44^a^	1.48 ± 0.04^a^	32.34 ± 0.98^b^
	Aging model group	63.99 ± 4.20^b^	1.35 ± 0.06^b^	20.67 ± 2.16^c^
	Vc positive control group	100.36 ± 5.01^a^	1.59 ± 0.05^a^	48.11 ± 2.17^a^
	Extract treatment group	95.58 ± 4.05^a^	1.61 ± 0.06^a^	46.85 ± 1.81^a^
Liver	Negative control group	55.65 ± 2. 81^b^	0.84 ± 0. 13^b^	54.61 ± 3. 32^a^
	Aging model group	49.54 ± 2. 88^c^	0.74 ± 0. 12^c^	45.72 ± 4. 51^b^
	Vc positive control group	59.75 ± 3.89^a^	1.24 ± 0.31^a^	56.18 ± 4.38^a^
	Extract treatment group	60.98 ± 4. 09^a^	1.12 ± 0. 29^a^	55.16 ± 5. 09^a^
Serum	Negative control group	86.42 ± 9. 29^b^	40.52 ± 3.32^b^	0.31 ± 0.08^a^
	Aging model group	72.58 ± 6.47^c^	33.76 ± 4.51^c^	0.12 ± 0.013^b^
	Vc positive control group	96.97 ± 7.87^a^	47.55 ± 3.69^a^	0.34 ± 0.06^a^
	Extract treatment group	98.65 ± 8.00^a^	47.21 ± 3.24^a^	0.35 ± 0.057^a^
Heart	Negative control group	51.58 ± 2.96^a^	0.51 ± 0.06^a^	5.06 ± 0.75^a^
	Aging model group	33.96 ± 2.19^c^	0.26 ± 0.05^b^	1.53 ± 0.38^b^
	Vc positive control group	42.87 ± 1.89^b^	0.54 ± 0.07^a^	5.01 ± 0.54^a^
	Extract treatment group	44.75 ± 2.45^b^	0.48 ± 0.09^a^	4.91 ± 0.45^a^
Kidney	Negative control group	56.24 ± 4.57^b^	1.76 ± 0.27^a^	14.30 ± 1.34^b^
	Aging model group	50.87 ± 5.09^c^	1.51 ± 0.14^b^	5.24 ± 1.06^c^
	Vc positive control group	63.77 ± 5.31^a^	1.68 ± 0.15^a^	17.65 ± 1.87^a^
	Extract treatment group	61.33 ± 6.47^a^	1.79 ± 0. 20^a^	18.73 ± 1. 61^a^

Note: Data were expressed as mean ± SD; One-way analysis of variance (ANOVA) was used to analyze the difference among groups; Values with different letters showed significant difference

*SOD* superoxide dismutase, *NOS* nitric oxide synthase, *CAT* catalase

**Table 3:** Effects of *Asparagus cochinchinensis* (Lour.) Merr on the NO and MDA contents

	Treatment	NO/μmol L^−1^	MDA/U mg^−1^ prot
Brain	Negative control group	12.50 ± 1.06^b^	1.43 ± 0.089^b^
	Aging model group	5.08 ± 0.850^c^	2.59 ± 0.25^a^
	Vc positive control group	26.36 ± 1.89^a^	0.89 ± 0.034^c^
	Extract treatment group	24.34 ± 1.83^a^	1.05 ± 0.028^c^
Liver	Negative control group	4.95 ± 0.17^b^	1.12 ± 0.27^b^
	Aging model group	1.91 ± 0.16^c^	1.55 ± 0.14^a^
	Vc positive control group	5.57 ± 0.36^a^	0.98 ± 0.11^c^
	Extract treatment group	5.64 ± 0.31^a^	1.10 ± 0.20^b^
Serum	Negative control group	907.64 ± 46.14^b^	24.96 ± 3.80^b^
	Aging model group	503.26 ± 27.08^c^	29.64 ± 4.46^a^
	Vc positive control group	987.89 ± 51.27^a^	21.87 ± 3.14^c^
	Extract treatment group	965.52 ± 41.44^a^	22.97 ± 2.81^c^
Heart	Negative control group	4.08 ± 0.92^a^	0.39 ± 0.078^b^
	Aging model group	1.10 ± 0.24^c^	2.53 ± 0.35^a^
	Vc positive control group	3.77 ± 0.44^b^	0.36 ± 0.08^b^
	Extract treatment group	3.61 ± 0.38^b^	0.45 ± 0.08^b^
Kidney	Negative control group	6.12 ± 0.62^b^	2.24 ± 0.39^b^
	Aging model group	3.09 ± 0.27^c^	4.90 ± 0.51^a^
	Vc positive control group	12.66 ± 1.45^a^	1.69 ± 0.49^c^
	Extract treatment group	13.21 ± 1.67^a^	1.73 ± 0.45^c^

Note: Data were expressed as mean ± SD; One-way analysis of variance (ANOVA) was used to analyze the difference among groups; Values with different letters showed significant difference

*NO* nitric oxide, *MDA* malondialdehyde

### Effect of the aqueous root extract of *A. cochinchinensis* on the microstructure of mouse viscera

**Fig. 2:**
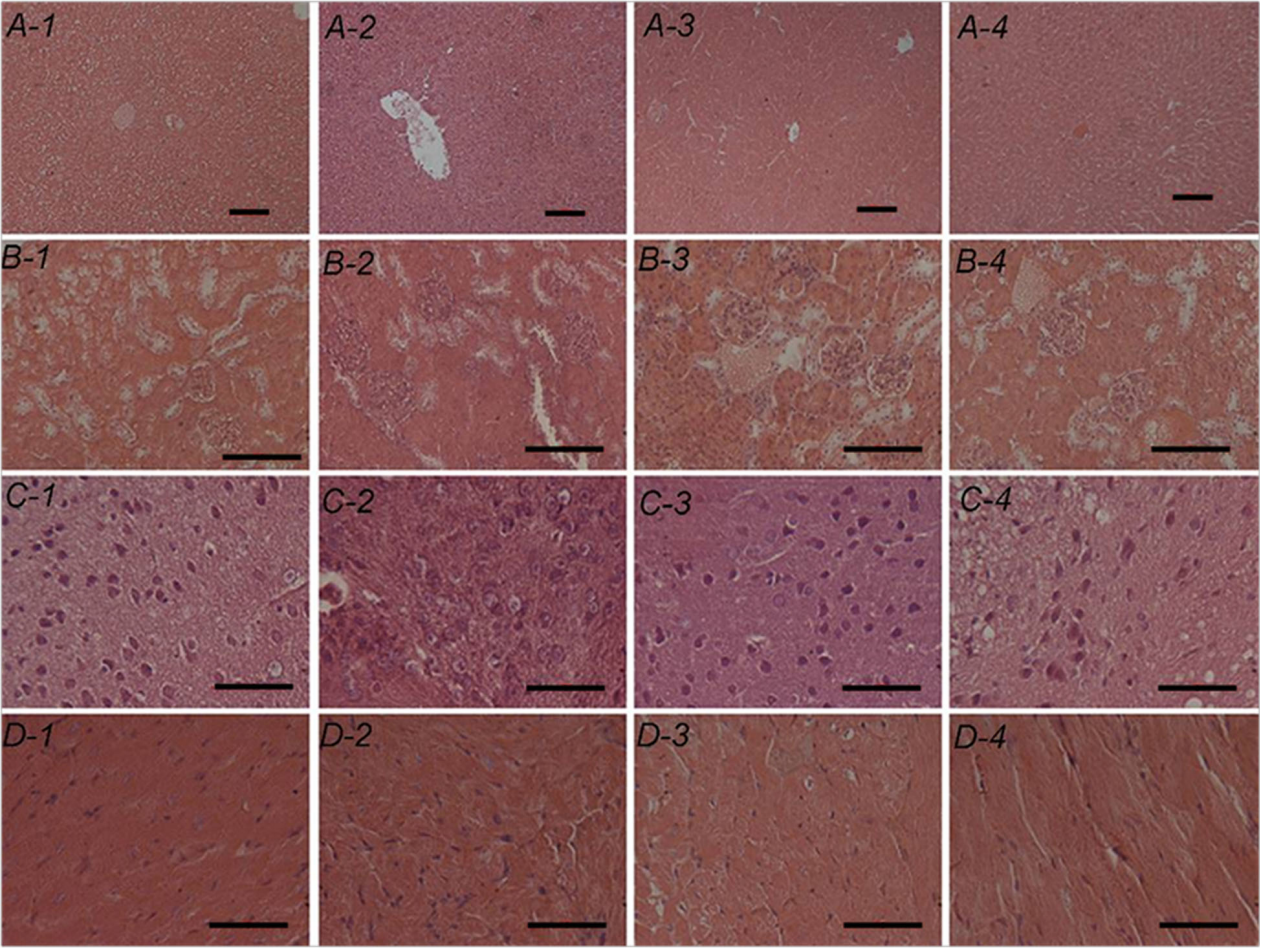
Different tissues stained with hematoxylin and eosin obtained from D-galactose-induced senile mice. A, liver; B, kidney; C, brain; D, heart; 1, the control group; 2, the aging model group; 3, the Vc control group; and 4, the extract treatment group. *Scale bars*: 100 μm (liver and kidney) or 50 μm (brain and heart)

HE results of tissues are shown in Fig. 2. In the control group, myocardial fiber cells were shuttle-shaped and arranged in parallel; had clear intercellular boundaries, close packing, clear visible band and intercalated disc; and distinct gradation in kidney, heart, brain, lung, and liver tissues. However, plump myocardial fiber cells, clear capillary vessels, and a widened interval were found in these tissues in the aging model group. After treatment with the aqueous root extract of *A. cochinchinensis* or Vc, a clear improvement in mouse viscera was observed. However, the aqueous root extract of *A. cochinchinensis* showed protective effects on the liver, brain, and kidney.

### Transcriptional gene expression levels related to antioxidation

**Fig. 3:**
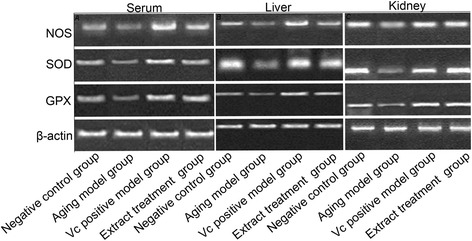
Nitric oxide synthase (NOS), superoxide dismutase (SOD), and glutathione peroxidase (GPX) gene expression levels in the serum, liver, and kidney, as determined by semi-quantitative reverse-transcription polymerase chain reaction

As shown in Fig. 3, compared with the control group, NOS, SOD, and GPX expression levels were significantly reduced in the aging group. However, compared with the aging model group, NOS, SOD, and GPX expression levels in the serum after treatment with root extract and Vc were elevated. NOS and GPX gene expression levels were increased in the extract treatment group compared with those in the negative control group. Moreover, NOS, SOD, and GPX gene expression levels in the liver of the negative control group was similar to those in the liver of the extract treatment group. In addition, NOS, SOD, and GPX gene expression levels in the kidney were similar among the control, Vc, and extract treatment groups.

## Discussion

The present study investigated the mechanism of action of the natural antioxidant *A. cochinchinensis* in the aging process. The results revealed that the aqueous root extract of *A. cochinchinensis* showed strong radical scavenging ability in vitro and could also increase *SOD* and *GPX* expression levels and SOD and CAT activities in vivo. Moreover, the aqueous root extract of *A. cochinchinensis* reduced the MDA content, increased the NO content, and played important roles in pathological changes in the liver, kidney, and brain.

A previous study showed that radicals could cause all kinds of diseases related to oxidative damage, such as cancer, cardiovascular disease, immune system defects, and aging [19]. Natural substances have been considered to inhibit radical-caused damage [20] and radical production [21]. MDA, as a peroxide product of lipids, could reflect oxygen radical production [22]. SOD could reduce MDA production by inducing a disproportionation reaction of superoxide radicals [23]. CAT, as an antioxidant enzyme, was also reported to influence peroxide hydrogen production [24]. GPX was also reported to reduce oxidative stress [25]. This study found that the aqueous root extract of *A. cochinchinensis* showed radical scavenging ability in vitro and could significantly influence SOD, CAT, GPX, and MDA activities or contents in vivo, indicating that the radical scavenging ability of aqueous root extract might be mediated by antioxidant enzymes.

Moreover, we found that *NOS* gene expression and NO content were upregulated in aging mice after been treated with the aqueous root extract of *A. cochinchinensis*. NO is produced from L-arginine by NOS, and reduced NOS activity could result in aging [26]. Therefore, the aqueous root extract of *A. cochinchinensis* might promote antioxidant activity by enhancing NOS expression and NO content. Previous studies also indicated that NO was associated with a series of physiological processes and NO could enhance the antioxidant activity [27, 28]. In addition, the overexpressed NO might cause aging by inducing toxicity of superoxide anions [29, 30]. Thus, we speculated that the aqueous root extract of *A. cochinchinensis* might influence NO production and then delay the aging process by destroying homeostasis.

## Conclusions

In conclusion, the antioxidant ability of the aqueous root extract of *A. cochinchinensis* was shown to occur through enhancement of antioxidant enzyme expression levels, increase in NOS, CAT, and SOD activities and the NO content, and reduction in the MDA content. However, chemical analysis of the extract is still required for identifying its active ingredient.

## Abbreviations

ABTS^+^: 3-ethylbenzothiazoline-6-sulfonic
CAT: Catalase
DPPH: 1,1-diphenyl-2-picrylhydrazyl radical
GPX: Glutathione peroxidase
KM: KunMing
MDA: Malondialdehyde
NO: Nitric oxide
NOS: Nitric oxide synthase
OH: Hydroxyl radical
SOD: Superoxide dismutase
Vc: Vitamin C
